# Early postoperative changes of sphingomyelins and ceramides after laparoscopic sleeve gastrectomy

**DOI:** 10.1186/s12944-018-0917-z

**Published:** 2018-11-24

**Authors:** Hakan Özer, İbrahim Aslan, Mehmet Tahir Oruç, Yaşar Çöpelci, Ebru Afşar, Sabriye Kaya, Mutay Aslan

**Affiliations:** 1 Internal Medicine Clinic, Antalya Research and Education Hospital, Antalya, Turkey; 2 Endocrinology Clinic, SBU Antalya Research and Education Hospital, Antalya, Turkey; 3 Surgery Clinic, Antalya Research and Education Hospital, Antalya, Turkey; 4 Department of Medical Biochemistry, Akdeniz University Medical Faculty, 07070 Antalya, Turkey

**Keywords:** Laparoscopic sleeve gastrectomy, Sphingomyelin, Ceramide

## Abstract

**Background:**

This study aimed to determine early postoperative changes of serum sphingomyelin (SM) and ceramide (CER) species following laparoscopic sleeve gastrectomy (LSG).

**Methods:**

Twenty obese patients [mean body mass index (BMI) 45,64 ± 6,10 kg/m^2^] underwent LSG and normal weight control patients (mean BMI 31,51 ± 6,21 kg/m^2^) underwent laparoscopic cholecystectomy. Fasting blood samples were collected prior to surgery, at day 1 and day 30 after surgery. Circulating levels of C16-C24 SMs, C16-C24 CERs and sphingosine-1-phosphate (S1P) were determined by an optimized multiple reaction monitoring (MRM) method using ultra fast-liquid chromatography (UFLC) coupled with tandem mass spectrometry (MS/MS). Serum activity of neutral sphingomyelinase (N-SMase) was assayed by standard kit methods, and ceramide-1-phosphate (C1P) levels were determined by enzyme-linked immunosorbent assay (ELISA). Lipid profile, routine biochemical and hormone parameters were assayed by standard kit methods. Insulin sensitivity was evaluated using homeostatic model assessment for insulin resistance (HOMA IR).

**Results:**

A significant decrease was observed in serum levels of very-long-chain C24 SM, very-long-chain C22-C24 CERs, HOMA-IR, N-SMase and C1P in LSG patients after postoperation day 1 and day 30 compared to preoperation levels. At 30 days postsurgery, BMI was reduced by 11%, fasting triglycerides were significantly decreased, and insulin sensitivity was increased compared to presurgery values. A significant positive correlation was found between HOMA-IR and serum levels of C22-C24 CERs in LSG patients.

**Conclusion:**

We conclude that very long chain CERs may mediate improved insulin sensitivity after LSG.

## Introduction

Morbid obesity has reached epidemic proportions in many societies [1]. Obesity is associated with increased risk of type 2 diabetes mellitus (T2DM), insulin resistance, and cardiovascular disease (CVD) [2]. Bariatric surgery offers morbidly obese individuals significant weight loss and a reduction of obesity-related comorbidities [3]. Laparoscopic sleeve gastrectomy (LSG) is a restrictive approach to the surgical management of morbid obesity. In LSG, subtotal gastric resection of the fundus and body creates a narrow, tubular stomach [3]. Recent reports have shown that LSG not only reduces the volume of the stomach but is also associated with a high rate of resolution of T2DM and other obesity associated comorbidities such as insulin resistance and hyperlipidemia [4].

The lipid pathways and the molecular mechanisms that control obesity-induced comorbidities are still subjects of intense research [5]. Studies have focused on the changes in lipid subfractions after LSG and have reported significant improvements in lipid profile changes in severely obese patients at 1 year after surgery [6]. Sphingomyelins (SMs) are among the most abundant lipids in the circulation and alterations in SM metabolism have also been linked to obesity [7].

Ceramides (CERs) serve as precursors for SMs, which are bioactive hydrophobic backbones of SMs [8]. Plasma ceramides are elevated in obese T2DM patients and the increase correlates with the degree of insulin resistance and inflammation [9]. A persistent lipid oversupply results in excessive ceramide accumulation in muscles of obese individuals with T2DM [10, 11]. Ceramide accumulation within human tissues inhibits insulin action and subsequent glucose uptake through inactivation of *protein kinase B* (*PKB*), also known as *Akt* [12]. Formation of CERs also lead to the production of metabolites such as ceramide-1-phosphate (C1P), sphingosine and sphingosine-1-phosphate (S1P) which are key regulators of inflammation [13]. During high fat diet-induced obesity, macrophages are activated by ceramides and promote adipose tissue inflammation in an NLRP3 (nucleotide-binding domain, leucine-rich-containing family, pyrin domain-containing-3) inflammasome-dependent manner [14]. Upregulation of the NLRP3 inflammasome together with high ceramide content in the plasma and subcutaneous adipose tissue of obese adolescents also contributes to the development of insulin resistance [14]. Inhibition of ceramide synthesis markedly improves glucose tolerance and prevents the onset of frank diabetes in obese rodents [15].

In connection with their possible involvement in insulin resistance and inflammation, changes in plasma sphingolipid levels after LSG, may serve as a biomarker of insulin resistance and inflammation. The aim of this study was to quantify individual SMs and CERs in the circulation of severely obese patients undergoing LSG; at baseline, day 1 and day 30 postoperatively. We also examined the relationship between plasma ceramide levels and insulin resistance as well as circulating N-SMase activity, C1P, S1P at all time-points.

## Materials and methods

### Study groups

The sleeve gastrectomy group included 20 obese female patients who were admitted to Antalya Research and Education Hospital, Surgery Clinic. The mean body mass index (BMI) of patients was 45,64 ± 6,10 kg/m^2^. All patients went through a clinical, biochemical, and pre-anesthetic evaluation, and subjects with apparent history of stroke, coronary heart disease, arrhythmia, peripheral artery disease, severe kidney dysfunction, liver disease, thyroid dysfunction, and infectious disease were excluded. All patients met the surgical indication criteria in the inter-disciplinary European guidelines on surgery of severe obesity [16]. Fasting blood samples were collected from all sleeve gastrectomy patients prior to surgery, at day 1 and day 30 after surgery. The control group included 15 age and gender matched patients who were admitted to Antalya Research and Education Hospital, Surgery Clinic. Patients in the control group underwent laparoscopic abdominal surgery for cholecystectomy. Subjects with apparent history of stroke, coronary heart disease, arrhythmia, peripheral artery disease, severe kidney dysfunction, liver disease, thyroid dysfunction, and infectious disease were excluded. The mean body mass index of patients in the control group was 31,51 ± 6,21 kg/m^2^ and all were non-smokers. Fasting blood samples were collected from the control groups prior to surgery, at day 1 and day 30 after surgery. All sleeve gastrectomy patients and control group subjects gave written informed consent prior to entry. This study was approved by the Institutional Review Board of Antalya Research and Education Hospital and was performed in accordance with the Declaration of Helsinki.

### Laboratory measurements

Hemoglobin (Hb) levels were measured on LH 780 Hematology Analyzer (Beckman Coulter, CA, USA). Serum glucose, insulin, alanine aminotransferase (ALT), alkaline phosphatase (ALP), creatine phosphokinase (CPK), creatinine, uric acid, total cholesterol (TC), high-density lipoprotein cholesterol (HDL-C) and triacylglycerol (TG) were assayed by standard kit methods using an auto-analyzer (Beckman Coulter AU5800, CA, USA). Low-density lipoprotein cholesterol (LDL-C) and very low-density lipoprotein cholesterol (VLDL-C) levels were calculated via the Friedewald formula [17]. Insulin sensitivity was evaluated using homeostatic model assessment for insulin resistance (HOMA IR) [18]. Serum ferritin, vitamin B12, vitamin D and thyroid-stimulating hormone (TSH) were assayed by standard kit methods using an auto-analyzer (Beckman Coulter DxI 800). Hemoglobin A1c (HbA1c) levels were measured on an automated glycohemoglobin Analyzer HLC-723G7 (Tosoh Bioscience, Tokyo, Japan).

### Measurement of serum sphingomyelins and ceramides

An optimized MRM method was developed using UFLC coupled with MS/MS as previously described [19]. Standards for sphingosine-1-phosphate (S1P), N-palmitoyl-D-erythro-sphingosylphosphorylcholine (C16 SM), N-stearoyl-D-erythro sphingosylphosphorylcholine (C18 SM), N-lignoceroyl-D-erythro sphingosylphosphorylcholine (C24 SM) N-palmitoyl-D-erythro-sphingosine (C16 CER), N-stearoyl-D-erythro-sphingosine (C18 CER), N-arachidoyl-D-erythro-sphingosine (C20 CER), N-behenoyl-D-erythro-sphingosine (C22 CER) and N-lignoceroyl-D-erythro-sphingosine (C24 CER) were purchased from Avanti Polar Lipids (Alabaster, AL, USA). Labeled C16 CER d18:1/16:0 (Palmitoyl-U-13C16) internal standard was obtained from Cambridge Isotope Laboratories (Andover, MA, USA). Solutions of each sphingolipid standard was prepared in methanol (Sigma-Aldrich, St. Louis, MO, USA) at 40 °C with sonication. A UFLC system (LC-20 AD UFLC XR, Shimadzu Corporation, Japan) was coupled to a LCMS-8040 triple quadrupole mass spectrometer (Shimadzu Corporation, Japan). Chromatographic separations were carried out using an HPLC column (XTerra C18, 2.1 mm X 50 mm, Waters, MA, USA) maintained at 60 °C. Sphingolipids were separated using a gradient elution with a flow rate of 0.45 ml/min. Mobile phase solvent A was water–acetonitrile–2-propanol (8:1:1, *v*/*v*/v) with 10 mM ammonium formate and solvent B was acetonitrile–2-propanol (9:1, v/v). Gradient program was solvent B, 65% (0–2 min), 90% (2.01–13 min), 100% (13.01–20 min) and 65% (20.1–23 min). MRM transitions and responses were automatically optimized for individual compounds in positive electrospray ionization (ESI). In the positive ESI-MS mode the precursor and product m/z values for all analyzed sphingolipids were as follows: S1P, precursor m/z: 380.10, product m/z: 264.40; C16 SM, precursor m/z: 703.30, product m/z: 184.20; C18 SM, precursor m/z: 731.40, product m/z: 184.20; C24 SM, precursor m/z: 815.50, product m/z: 184.20; C16 CER, precursor m/z: 538.50, product m/z: 264.40; C16 CER *IS, precursor m/z: 554.30, product m/z: 264.30; C18 CER, precursor m/z: 566.30, product m/z: 264.40; C20 CER, precursor m/z: 594.60, product m/z: 264.50; C22 CER, precursor m/z: 622.60, product m/z: 264.40; C24 Ceramide, precursor m/z: 650.40, product m/z: 264.30. Responses to analyzed sphingolipids were optimized to a linear calibration range from 39 to 625 ng/ml and a sample analysis time of 23 min.

### Preparation of serum samples for mass spectrometric analysis

Serum sphingolipid extraction was done as previously described [20]. Two μl of 5 μg/ml internal standard solution was added to 500 μl of serum sample 1:20 (*v*/v) diluted in distilled water. Samples were briefly vortexed and 375 μl of chloroform/methanol (1:2, v/v) was added. The samples were sonicated for 30 sn and 100 μl of water was added. The mixtures were vortexed for 5 min and stood at room temperature for 30 min. After centrifugation at 2000 g for 5 min, the supernatant was taken and 125 μl of chloroform and 125 μl of water was added. The samples were vortexed and stood for 30 min for phase separation. The upper organic layer was transferred to glass tubes and evaporated at room temperature under a constant stream of nitrogen with height adjustable gas distribution unit (VLM, Bielefeld, Germany). The dried residue was dissolved in 100 μl of methanol and 10 ul was injected into the column.

### Measurement of Ceramide-1-phosphate levels

Ceramide-1-phosphate levels were measured in serum via an ELISA kit (MyBioSource, Catalog # MBS2601367, CA, USA). Serum C1P captured by a solid phase human monoclonal antibody was detected with a biotin-labeled polyclonal antibody. An avidin-peroxidase conjugate was then added to bind the biotinylated antibody. A TMB substrate was added and the yellow product was measured at 450 nm. A standard curve of absorbance values of known C1P standards was plotted as a function of C1P standard concentrations using the GraphPad Prism Software program for windows version 5.03. (GraphPad Software Inc.). The amount of C1P in the samples were calculated from their corresponding absorbance values via the standard curve.

### Measurement of neutral sphingomyelinase activity

Neutral-SMase activity was measured in serum via a SMase assay kit (Abcam, Catalog # ab138876, Cambridge, UK). The kit uses a colorimetric probe to quantify phosphocholine produced from the hydrolysis of SM. The generated color is measured colorimetrically at 655 nm. A standard curve of absorbance values of known amounts of SMase standards was generated. Sphingomyelinase activity in the samples (mU/dL) was calculated from their corresponding absorbance values via the standard curve.

### Statistical analysis

Statistical analysis was performed using SigmaStat statistical software version 2.0. Statistical analysis for each measurement is described in the figure and table legends. A *p* value of < 0,05 was considered statistically significant.

## Results

### Control and sleeve gastrectomy group characteristics

The LSG was composed of women. The mean ± SD of age, body weight, body mass index, was 37,2 ± 11,7 years, 114,6 ± 22,9 kg, 45,6 ± 6,1 kg/m^2^, respectively. The control group was also composed of women. The mean ± SD of age, body weight, and body mass index was 39,4 ± 12,7 years, 75,2 ± 16,2 kg, and 31,0 ± 6,2 kg/m^2^, respectively. Body mass index in the LSG group was significantly greater compared to the control group at preoperation and 30 days after operation (Table 1). There was a significant decrease in BMI in LSG patients at post operation day 30 compared to preoperation levels.

**Table 1:** Laboratory values in control and sleeve gastrectomy group

Control Group (*n = 15*)				Sleeve Gastrectomy Group (*n = 20*)		
Variable	Preop	Po Day 1	Po Day 30	Preop	Po Day 1	Po Day 30
BMI (kg/m^2^)	31,51 ± 6,21	ND	31,42 ± 5,90	45,64 ± 6,10^z^	ND	40,44 ± 6,53^c, r^
Hb (g/dl)	12,40 ± 1,34	12,02 ± 1,56	12,59 ± 1,31	11,76 ± 1,40	11,04 ± 1,16^a,x^	12,65 ± 1,31^a, b^
HbA1c (%)	5,67 ± 0,56	ND	5,87 ± 0,69	5,96 ± 0,38	ND	5,54 ± 0,43^c^
Glucose (mg/dl)	104,00 ± 36,52	95,50 ± 23,00	94,79 ± 15,79	104,10 ± 54,65	86,50 ± 27,71	86,10 ± 10,12
HOMA-IR	3,34 ± 2,92	2,62 ± 2,53	2,97 ± 2,31	5,27 ± 8,70	1,50 ± 1,25^c^	1,42 ± 0,80^c, y^
ALT (U/L)	34,38 ± 24,42	34,17 ± 24,00	23,71 ± 9,38	21,20 ± 10,93	38,73 ± 20,99^a^	28,15 ± 22,35
ALP (U/L)	87,21 ± 45,50	99,00 ± 62,75	84,64 ± 19,17	71,90 ± 17,27	69,73 ± 14,67	71,00 ± 17,43^q^
CPK (U/L)	44,31 ± 29,88	97,73 ± 87,73^m^	78,50 ± 31,46^m^	133,59^s^	106,07 ± 87,87	86,00 ± 39,70
Creatinine (mg/dl)	0,80 ± 0,14	0,79 ± 0,10	0,75 ± 0,06	0,81 ± 0,15	0,81 ± 0,12	0,86 ± 0,12
Uric Acid (mg/dl)	4,29 ± 1,20	4,80 ± 1,40	4,94 ± 0,99	5,79 ± 1,09^z^	5,67 ± 1,03	7,16 ± 2,27^d, e,y^
Ferritin (ng/ml)	68,79 ± 77,81	78,15 ± 75,12	37,71 ± 29,30	22,35 ± 25,12	49,07 ± 44,09	44,40 ± 83,51
Vitamin B12 (pg/ml)	279,64 ± 109,67	282,15 ± 94,72	241,07 ± 69,26	233,75 ± 126,52	336,53 ± 117,30^f^	300,95 ± 172,74
Vitamin D (ng/ml)	13,07 ± 6,96	15,31 ± 7,22	18,60 ± 10,55	13,81 ± 7,70	15,79 ± 8,23^a^	22,17 ± 10,84^d, e^
TSH (mU/l)	1,88 ± 1,30	ND	1,72 ± 0,60	3,05 ± 1,64	ND	2,12 ± 1,72^a^
TC (mg/dl)	182,00 ± 35,65	174,77 ± 36,33	172,36 ± 32,18	182,95 ± 48,73	164,00 ± 31,45	162,30 ± 46,33
HDL-C (mg/dl)	41,14 ± 15,02	39,85 ± 12,71	43,29 ± 6,59	39,95 ± 8,91	35,20 ± 5,57^a^	40,40 ± 8,92
LDL-C (mg/dl)	110,07 ± 31,92	110,08 ± 31,04	104,00 ± 26,76	111,35 ± 30,21	99,73 ± 21,05	101,15 ± 37,02
VLDL-C (mg/dl)	30,21 ± 15,45	27,23 ± 10,43	35,79 ± 25,78	35,75 ± 34,27	25,80 ± 8,29	20,85 ± 6,68^c, d^
TG (mg/dl)	147,79 ± 63,44	136,54 ± 52,50	154,71 ± 71,73	179,05 ± 170,85	127,20 ± 40,65	104,85 ± 33,66^c,d^

Values are mean ± SD. *Preop* preoperation, *Po* postoperation, *ND* not determined, *BMI* body mass index, *Hb* hemoglobin, *HbA1c* hemoglobin A1c, *HOMA-IR* homeostasis model assessment of insulin resistance, *ALT* Alanine aminotransferase, *ALP* alkaline phosphatase, *CPK* creatine phosphokinase, *TSH* thyroid-stimulating hormone, *CRP* C-reactive protein, *TC* total cholesterol, *HDL-C* high-density lipoprotein cholesterol, *LDL-C* low-density lipoprotein cholesterol, *VLDL-C* very low-density lipoprotein cholesterol, *TG* triglyceride

a, *p* < 0,05 vs. sleeve gastrectomy Preop. Statistical analysis was done by paired t-test

b, *p* < 0,001 vs. sleeve gastrectomy Po day 1. Statistical analysis was done by paired t-test

c, *p* < 0,001 vs. sleeve gastrectomy Preop. Statistical analysis was done by Wilcoxon Signed Rank Test

d, *p* < 0,05 vs. sleeve gastrectomy Po day 1. Statistical analysis was done by paired t-test

e, *p* = 0,01 vs. sleeve gastrectomy Preop. Statistical analysis was done by paired t-test

f, *p* < 0,001 vs. sleeve gastrectomy Preop. Statistical analysis was done by paired t-test

m, *p* < 0,05 vs. control Preop. Statistical analysis was done by paired t-test

q, *p* < 0,05 vs. control Po day 30. Statistical analysis was done by t-test

r, *p* = < 0,001 vs. control Po day 30. Statistical analysis was done by t-test

s, *p* = 0,001 vs. control Preop. Statistical analysis was done by Mann-Whitney Rank Sum Test

x, *p* = 0,042 vs. control Po day 1. Statistical analysis was done by t-test

y, *p* = 0,005 vs. control Po day 30. Statistical analysis was done by Mann-Whitney Rank Sum Test

z, *p* = < 0,001 vs. control Preop. Statistical analysis was done by t-test

### Biochemical measurements

Laboratory values measured in LSG patients and control group are shown in Table 1. Hemoglobin levels were significantly decreased at post operation day 1 in LSG patients compared to preoperation levels (*p* < 0,05). This decrease caused a significant difference in Hb levels between LSG and control group patients at post operation day 1 (*p* = 0,042). Hemoglobin levels were significantly restored in LSG patients at post operation day 30. Homeostasis model assessment of insulin resistance showed a significant decrease in LSG patients at post operation day 1 and day 30, compared to preoperation levels (*p* < 0,001). The HOMA-IR levels in LSG patients at post operation day 30 were also significantly lower than the control group (*p* = 0,005). ALT levels showed a significant increase in LSG patients at post operation day 1 compared to preoperation levels (*p* < 0,05). Preoperation levels of CPK was significantly greater in LSG patients compared to the control group (*p* = 0,001). Creatine phosphokinase levels significantly increased in the control group following cholecystectomy (*p* < 0,05). Preoperation and post operation day 30 levels of uric acid were significantly higher in LSG patients compared to the control group (*p* < 0,001). Uric acid levels significantly increased in LSG patients at post operation day 30 compared to both preoperation and post operation day 1. 25-(OH)-vitamin D levels significantly increased following LSG. Post operation day 30 levels of 25-(OH)-vitamin D were significantly greater than both preoperation and post operation day 1. (*p* < 0,05). HDL-C levels significantly decreased in LSG patients at postoperation day 1 compared to preoperation levels (p < 0,05). A significant decrease was observed in TG and VLDL-C levels in LSG patients at postoperation day 30 compared to both preoperation and post operation day 1 (*p* < 0,001 and *p* < 0,05, respectively).

### Serum sphingomyelin levels

**Fig. 1:**
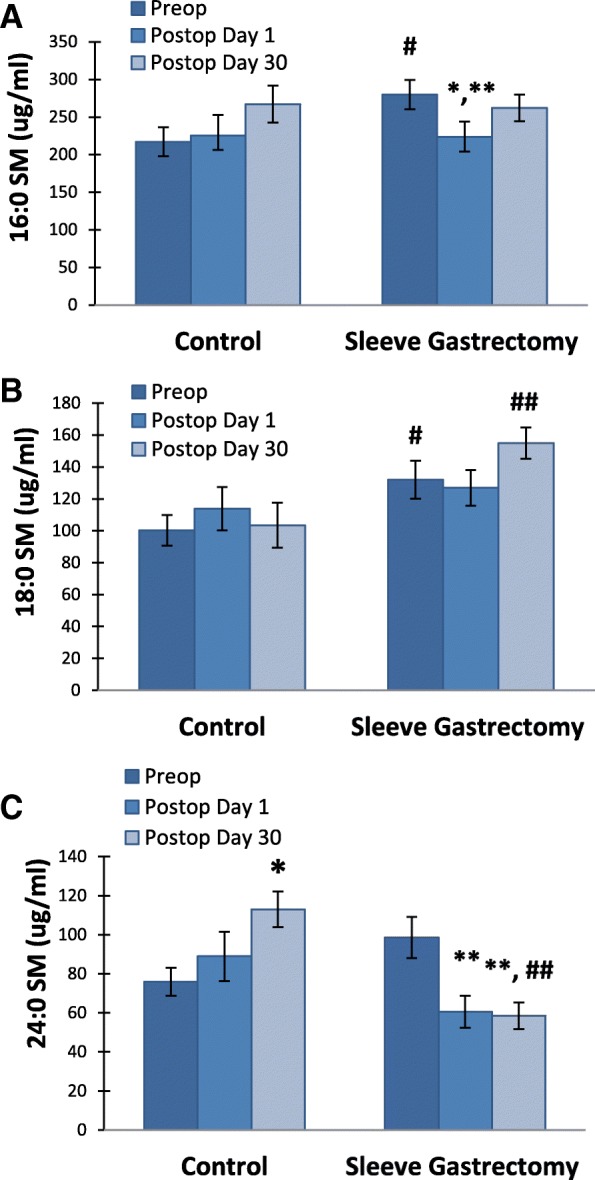
Serum sphingomyelin levels. Values measured in control group (*n* = 15) and LSG patients (*n* = 20). All values are mean ± SEM. SM, sphingomyelin. **a** 16:0 SM (d18:1/16:0). #, *p* < 0,05 vs. control Preop. Statistical analysis was done by t-test. *, *p* = 0,015 vs. sleeve gastrectomy Preop. Statistical analysis was done by paired t-test. **, *p* = 0,006 vs. sleeve gastrectomy Po day 30. Statistical analysis was done by paired t-test. **b** 18:0 SM (d18:1/18:0). #, *p* < 0,05 vs. control Preop. Statistical analysis was done by t-test. ##, *p* < 0,01 vs. control Po day 30. Statistical analysis was done by t-test. **c** 24:0 SM (d18:1/24:0). *, *p* < 0,001 vs. control Preop. Statistical analysis was done by paired t-test. **, *p* < 0,001 vs. sleeve gastrectomy Preop. Statistical analysis was done by paired t-test. #, *p* < 0,01 vs. control Po day 30. Statistical analysis was done by t-test

Serum levels of SMs are given in Table 2. Preoperation levels of C16 SM were significantly greater in LSG patients compared to the control group (*p* < 0,05) (Fig. 1 a). A significant decrease was observed in C16 SM levels in LSG patients at postoperation day 1 compared to both preoperation and post operation day 30 (*p* = 0,015 and p = 0,006, respectively). C16 SM levels were restored in LSG patients at post operation day 30. Preoperation and post operation day 30 levels of C18 SM were also significantly higher in LSG patients compared to the control group (*p* < 0,05 and p < 0,01, respectively) (Fig. 1 b). Long chain C24 SM levels significantly decreased in LSG patients after surgery (Fig. 1 c). This decrease was evident both at post operation day 1 and day 30 (*p* < 0,001). Unlike the LSG group, C24 SM levels increased in the control group following cholecystectomy. This increase was significant at postoperation day 30 (*p* < 0,001).

**Table 2:** Levels of serum sphingomyelins

Control Group (*n = 15*)				Sleeve Gastrectomy Group (*n = 20*)		
Sphingolipids (μg/ml)	Preop	Po Day 1	Po Day 30	Preop	Po Day 1	Po Day 30
16:0 SM (d18:1/16:0)	217,16 ± 19,10	225,40 ± 27,36	267,25 ± 24,50	279,88 ± 19,43^a^	223,68 ± 20,34^b,c^	262,14 ± 17,85
18:0 SM (d18:1/18:0)	100,24 ± 9,67	113,84 ± 13,51	103,51 ± 14,02	131,97 ± 11,91^a^	126,93 ± 11,10	154,90 ± 9,83^d^
24:0 SM (d18:1/24:0)	75,94 ± 7,19	88,93 ± 12,62	113,06 ± 9,16^e^	98,64 ± 10,59	60,57 ± 8,14^f^	58,54 ± 6,84^f,d^

Values are mean ± SEM. Preop, preoperation; Po, postoperation. 16:0 SM (d18:1/16:0), N-palmitoyl-D-erythrosphingosylphosphorylcholine; 18:0 SM (d18:1/18:0), N-stearoyl-D-erythro-sphingosylphosphorylcholine; 24:0 SM (d18:1/24:0), N-lignoceroyl-D-erythro-sphingosylphosphorylcholine

a, *p* < 0,05 vs. control Preop. Statistical analysis was done by t-test

b, *p* = 0,015 vs. sleeve gastrectomy Preop. Statistical analysis was done by paired t-test

c, *p* = 0,006 vs. sleeve gastrectomy Po day 30. Statistical analysis was done by paired t-test

d, *p* < 0,01 vs. control Po day 30. Statistical analysis was done by t-test

e, *p* < 0,001 vs. control Preop. Statistical analysis was done by paired t-test

f, *p* < 0,001 vs. sleeve gastrectomy Preop. Statistical analysis was done by paired t-test

### Serum ceramide levels

**Fig. 2:**
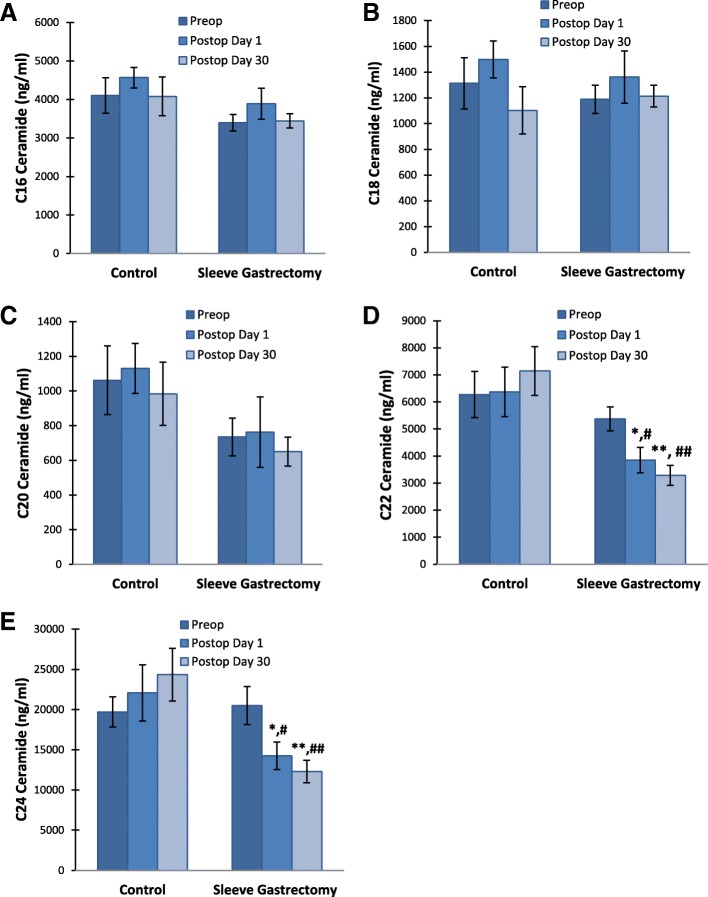
Serum ceramide levels. Values measured in control group (n = 15) and LSG patients (n = 20). All values are mean ± SEM. **a** C16 Ceramide (d18:1/16:0) **b** C18 Ceramide (d18:1/18:0) **(c)** C20 Ceramide (d18:1/20:0) **d** C22 Ceramide (d18:1/22:0).*, *p* = 0,014 vs. sleeve gastrectomy preop. Statistical analysis was done by paired t-test. #, p = 0,008 vs. control Po Day 1. Statistical analysis was done by t-test. **, *p* < 0,001 vs. sleeve gastrectomy preop. Statistical analysis was done by paired t-test. ##, p < 0,001 vs. control Po Day 30. Statistical analysis was done by t-test. **e** Serum levels of C24 Ceramide (d18:1/24:0). *, p = 0,005 vs. sleeve gastrectomy preop. Statistical analysis was done by paired t-test. #, *p* = 0,022 vs. control Po Day 1. Statistical analysis was done by t-test. **, *p* < 0,001 vs. sleeve gastrectomy preop. Statistical analysis was done by paired t-test. ##, *p* < 0,001 vs control Po Day 30. Statistical analysis was done by t-test

**Fig. 3:**
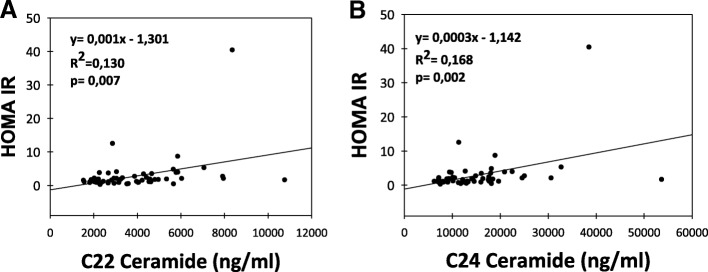
Correlation of HOMA IR with serum ceramide levels. **a** C22 Ceramide (d18:1/22:0). **b** C24 Ceramide (d18:1/24:0). Correlations were evaluated by linear regression analysis. A *p* value of < 0.05 was considered statistically significant

Serum levels of CERs are given in Table 3. No significant changes were observed in C16-, C 18- and C20-CER levels between and within the two groups (Fig. 2). Long-chain C22- and C24- CER levels were significantly decreased in LSG patients after postoperation day 1 and day 30 compared to preoperation levels (*p* < 0,05). Likewise, postoperation day 1 and day 30 C22- and C24- CER levels were significantly lower in LSG patients compared to controls (*p* < 0,05). A significant positive correlation was found between HOMA-IR and serum levels of C22-C24 CERs in LSG patients (Fig. 3).

**Table 3:** Levels of serum ceramides

Control Group (*n = 15*)				Sleeve Gastrectomy Group (*n = 20*)		
Ceramide (ng/ml)	Preop	Po Day 1	Po Day 30	Preop	Po Day 1	Po Day 30
C16 CER (d18:1/16:0)	4107,02 ± 461,12	4568,07 ± 265,51	4080,22 ± 503,81	3397,68 ± 217,02	3892,78 ± 401,89	3447,04 ± 183,08
C18 CER (d18:1/18:0)	1312,73 ± 198,25	1498,11 ± 144,02	1103,22 ± 182,85	1188,98 ± 109,14	1361,44 ± 203,41	1213,58 ± 84,05
C20 CER (d18:1/20:0)	1062,13 ± 145,50	1130,25 ± 125,52	983,98 ± 145,01	734,67 ± 79,31	762,52 ± 158,10	650,10 ± 77,73
C22 CER (d18:1/22:0)	6273,70 ± 851,29	6372,18 ± 916,66	7144,98 ± 899,62	5374,87 ± 440,21	3850,60 ± 471,05^a,b^	3287,03 ± 371,39^c,d^
C24 CER (d18:1/24:0)	19,706,45 ± 1892,46	22,071,44 ± 3507,56	24,340,62 ± 3289,63	20,507,48 ± 2366,78	14,240,44 ± 1709,18^e,f^	12,307,39 ± 1400,13^g,h^

Values are mean ± SEM. Preop, preoperation; Po, postoperation. C16 Ceramide (d18:1/16:0), N-palmitoyl-D-erythro-sphingosine; C18 Ceramide (d18:1/18:0), N-stearoyl-D-erythro-sphingosine; C20 Ceramide (d18:1/20:0), N-arachidoyl-D-erythro-sphingosine; C22 Ceramide (d18:1/22:0), N-behenoyl-D-erythro-sphingosine; C24 Ceramide (d18:1/24:0), N-lignoceroyl-D-erythro-sphingosine

a, *p* = 0,014 vs. sleeve gastrectomy preop. Statistical analysis was done by paired t-test

b, *p* = 0,008 vs. control Po Day 1. Statistical analysis was done by t-test

c, *p* < 0,001 vs. sleeve gastrectomy preop. Statistical analysis was done by paired t-test

d, *p* < 0,001 vs. control Po Day 30. Statistical analysis was done by t-test

e, *p* = 0,005 vs. sleeve gastrectomy preop. Statistical analysis was done by paired t-test

f, *p* = 0,022 vs. control Po Day 1. Statistical analysis was done by t-test

g, *p* < 0,001 vs. sleeve gastrectomy preop. Statistical analysis was done by paired t-test

h, *p* < 0,001 vs control Po Day 30. Statistical analysis was done by t-test

### Serum Sphingosine-1-phosphate levels

**Fig. 4:**
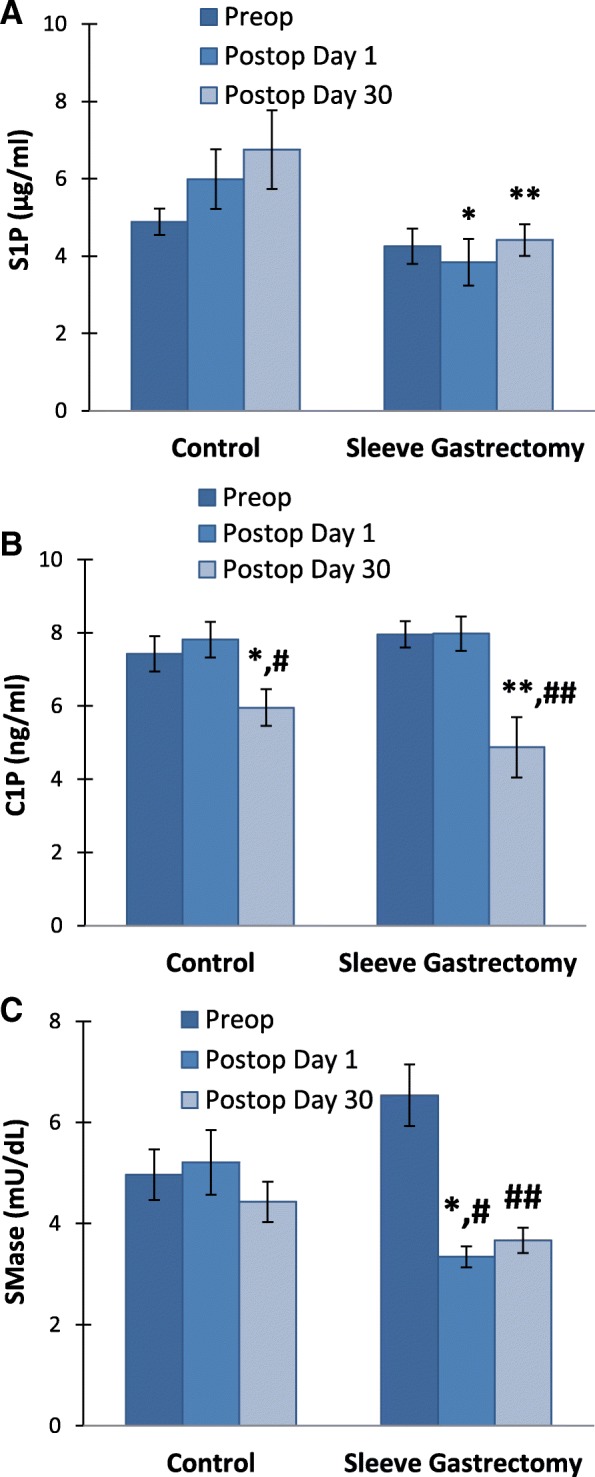
**a** Serum sphingosine 1 phosphate (S1P) levels. Values measured in control group (n = 15) and LSG patients (n = 20). All values are mean ± SEM.*, *p* = 0,013 vs. **, *p* = 0,029 vs. control postop day 30. Statistical analysis was done by Mann-Whitney Rank Sum Test. **b** Serum ceramide-1-phosphate (C1P) levels. Values measured in control group (n = 15) and LSG patients (n = 20). All values are mean ± SEM *, *p* = 0,013 vs. control preop. Statistical analysis was done by Wilcoxon Signed Rank Test. #, *p* = 0,020 vs. control postop day 1. Statistical analysis was done by paired t test. **, *p* = 0,004 vs. sleeve gastrectomy preop. Statistical analysis was done by Wilcoxon Signed Rank Test. ##, *p* = 0,005 vs. sleeve gastrectomy postop day 1. Statistical analysis was done by Wilcoxon Signed Rank Test. **c** Serum neutral sphingomyelinase (SMase) activity. . Values measured in control group (n = 15) and LSG patients (n = 20). All values are mean ± SEM. *, *p* = 0,021 vs. control postop day 1. Statistical analysis was done by Mann-Whitney Rank Sum Test. #, *p* < 0,001 vs. sleeve gastrectomy Preop. ##, *p* = 0,002 vs. sleeve gastrectomy Preop

Serum S1P levels (mean ± SEM) measured in both control group (*n* = 15) and sleeve gastrectomy patients (*n* = 20) at preoperation were 4,89 ± 0,34 and 4,25 ± 0,45 μg/ml respectively; at postoperation day 1 were, 5,99 ± 0,77 and 3,84 ± 0,60 μg/ml, respectively; and at postoperation day 30 were, 6,75 ± 1,02 and 4,41 ± 0,41 μg/ml, respectively. A significant decrease was observed in serum levels of S1P in LSG patients at postoperation day 1 and day 30 compared to controls (*p* < 0,05) (Fig. 4a).

### Serum Ceramide-1-phosphate levels

Serum C1P levels (mean ± SEM) measured in both control group (*n* = 15) and sleeve gastrectomy patients (*n* = 20) at preoperation were 7,43 ± 0,49 and 7,96 ± 0,36 ng/ml respectively; at postoperation day 1 were, 7,82 ± 0,49 and 7,97 ± 0,47 ng/ml, respectively; and at postoperation day 30 were, 5,96 ± 0,50 and 4,87 ± 0,83 ng/ml, respectively. A significant decrease was observed in serum levels of C1P in LSG patients and controls at postoperation day day 30 compared to preoperation levels and postoperation day 1 (*p* < 0,05) (Fig. 4b).

### Serum neutral sphingomyelinase activity

Preoperation levels of serum N-SMase activity (mean ± SEM) showed no significant difference between the control group (n = 15) and sleeve gastrectomy patients (n = 20) (4,96 ± 0,50 and 6,54 ± 0,61 mU/dL, respectively). A significant decrease was observed in serum levels of N-SMase in LSG patients (3,34 ± 0,21 mU/dL) compared to controls (5,21 ± 0,64 mU/dL) at postoperation day 1 (p < 0,05). Neutral SMase activity was significantly decreased in LSG patients after postoperation day 1 and day 30 compared to preoperation levels (*p* < 0,01) (Fig. 4 c).

## Discussion

To our knowledge, this is the first study evaluating early postoperative effects of LSG on serum sphingolipid levels. Plasma sphingolipids and ceramides play an important role in the pathogenesis of obesity-induced metabolic conditions and cardiovascular disease [21]. The role of sphingolipids in insulin sensitivity is relevant since CERs are important elements in insulin resistance and inflammation [22]. In this study we aimed to determine circulating levels C16-C24 SMs, C16-C24 CERs, S1P, C1P and neutral-SMase activity in serum obtained from LSG patients and controls.

We have observed 24,8 ± 14% of excess weight loss (EWL) at 30 days following LSG. Our results are in agreement with recent studies that reported 23,9 ± 9.6% [23] and 22 ± 2,21% [24] EWL at one month after LSG. There was a 5,2 ± 2,55 kg/m^2^ decrease in the BMI of LSG patients at 30 days following surgery (Table 1). This is in agreement with a recent study that reported an expected reduction of 5.65 kg/m^2^ BMI in LSG patients at 30 days after surgery [25]. We have also observed a significant reduction in insulin resistance occurring very early and at one month following sleeve gastrectomy. These findings are in agreement with studies reporting a significant decrease in insulin resistance both immediately after sleeve gastrectomy [26] and 30 days following LSG [27]. Improved insulin sensitivity in obese patients immediately after sleeve gastrectomy can be influenced by ghrelin, glucagon-like peptide 1 (GLP-1), and peptide YY (PYY) hormonal changes [28]. It is also important to note that decreased HOMA-IR levels observed soon after LSG is also not weight change-related.

Preoperation levels of C16 and C18 SM were significantly greater in LSG patients compared to the control group (Table 2 and Fig. 1). The first reaction of SM synthesis is catalyzed by serine palmitoyltransferase (SPT) and the rate of this reaction is influenced largely by the availability of free fatty acids [29]. Elevated systemic fatty acid availability therefore increases plasma SM levels in humans, suggesting that SM synthesis is driven by substrate availability [30]. The accumulation of short chain sphingolipids in obese patients is not likely to be caused by increased intake of sphingolipids since most injested forms are degraded in the gut by glucoceramidases, sphingomyelinases, and ceramidases [31]. Sphingolipids that resist degradation are secreted in the feces, mostly in the form of ceramide [31].

We have observed a significant decrease in serum levels of very-long-chain C24 SM and very-long-chain C22-C24 CERs in LSG patients after postoperation day 1 and day 30 compared to preoperation levels (Table 2 and Table 3). Ceramide synthesis via the de novo pathway involves ceramide synthase (CerS) enzymes that produce different ceramide species based on their fatty acyl chain length [32]. It has been reported that inhibition of ceramide synthesis ameliorates obesity-induced insulin resistance [32] and further suggested that altering the acyl chain composition of CERs may be a novel way of modulating insulin resistance [33].

It is known that bariatric surgery produces a marked decline in inflammatory cytokines secreted by adipose tissue such as tumor necrosis factor (TNF)-α, interleukin-1 (IL-1) and IL-6 [34] which induce the production of ceramide [35, 36]. Therefore, the decrease in serum levels of very-long-chain CERs in LSG patients following surgery (Fig. 2) may result from decreased inflammation. It has been reported that plasma TNF-α concentrations in obese type 2 diabetics correlate with long chain plasma ceramides [9]. It has also been shown that the decrease in long chain ceramide species correlated significantly with TNF-α levels at 6 months following gastric bypass surgery [37]. These findings further support the view that ceramides are associated with insulin resistance through inflammatory pathways.

It is important to acknowledge that the amount of circulating and tissue CERs depend on the equilibrium between production and degradation. Ceramide generation may result from hydrolysis of SM by SMase. Five types of SMase have been identified based on their cation dependence and pH optima of action [38]. Among the five types of SMase, the lysosomal acidic SMase and the magnesium-dependent neutral SMase are considered major candidates for the production of CERs in cellular response to inflammation [38]. Preoperation levels of serum N-SMase activity showed no significant difference between the control and sleeve gastrectomy patients (Fig. 4). These findings are in agreement with a recent study performed on human adipose tissue which showed that N-SMase activity did not change between control and obese patients [39]. Neutral SMase activity was significantly decreased in LSG patients after postoperation day 1 and day 30 compared to preoperation levels which can result in decreased formation CERs as shown in Table 3. To our knowledge this is the first study measuring circulating N-SMase activity following LSG. Decreased serum activity of N-SMase may contribute to decreased formation of circulating CERs. In fact, recent studies on cell cultures revealed that TNF-α can induce SMase activity, as well as increase ceramide levels [40]. Thus, it is likely that reduced N-SMase following LSG is related to decreased inflammation and can lead to decreased CER levels.

The main pathway of CER degradation is its deacylation by the enzyme ceramidase (CDase: acid, neutral, and alkaline) to yield free fatty acid (FFA) and sphingosine (SPH). SPH may be further phosphorylated to S1P by the enzyme SPH kinase. Formation of CERs also lead to the production of metabolites such as C1P, SPH and S1P which are key regulators of inflammation [13]. Previous work in humans have shown that plasma S1P positively correlated with total body fat percentage, BMI, waist circumference and HOMA-IR levels. [41]. To our knowledge this is the first study measuring circulating S1P levels following LSG. We have oberved no significant change in preoperation levels of S1P between control and sleeve gastrectomy patients, however a significant decrease was observed in serum levels of S1P in LSG patients at postoperation day 1 and day 30 compared to controls (*p* < 0,05) (Fig. 4). We found no significant correlation between HOMA-IR and serum levels of S1P.

We found a significant positive correlation between HOMA-IR and serum levels of C22-C24 CERs in LSG patients (Fig. 3). Recently, it was reported that Roux-en-Y gastric bypass surgery (RYGB) reduces plasma ceramide subspecies and improves insulin sensitivity in severely obese patients [37]. Data presented herein demonstrates reproducible decreases in plasma ceramide species early after LSG. A possible mechanism for the improvement in insulin sensitivity may be the significant reduction of long-chain ceramide supply which would restrain excessive ceramide accumulation in muscles of obese individuals [10, 11]. Studies with insulin-resistant human subjects showed abnormal ceramide accumulation in vastus lateralis muscle compared to lean subjects with no family history of diabetes [10]. It has also been shown that ceramide accumulation within human tissues inhibits insulin action and subsequent glucose uptake through inactivation of *protein kinase B* (*PKB*) [12]. Several groups have confirmed that ceramide promotes the dephosphorylation of Akt/PKB by protein phosphatase 2A [42]. An additional mechanism by which ceramide may impair insulin action is by assisting signaling pathways initiated by inflammatory cytokines, such as TNF-α, that activate serine/ threonine kinases known to impair insulin signaling [43]. The significant positive correlation between HOMA-IR and C22-C24 CER levels in LSG patients are coherent with results from cell culture studies which show that increased intracellular ceramide generation is associated with decreased insulin-stimulated glucose uptake, glycogen synthesis and Akt serine phosphorylation in C2C12 skeletal muscle cells [44]. Likewise, ceramide impairs the insulin-dependent membrane recruitment of protein kinase B leading to a loss in downstream signalling in skeletal muscle cells [45]. The effect of LSG on ceramide subspecies was noticeable for long chain C22 and C24 species. C24 CER, the most abundant species in human serum, is elevated in obese subjects with type 2 diabetes and plasma levels have been reported to correlate with the severity of insulin resistance [9]. Our data further show a significant positive correlation between insulin sensitivity and serum levels of C22-C24 CERs in LSG patients. In this context it is important to note that the observed correlation does not specifically imply a cause and effect. It is also critical to mention that we have measured ceramide subspecies in the serum rather than insulin targeted tissues such as skeletal muscle and adipose tissue. Other limitations of our work is that it covers a small cohort, it is nonrandomized study as patients were assigned to either group according to clinical criteria, and it only includes female patients. However, the limitation of gender distribution has been minimized by matching the control group similarly.

## Conclusions

In summary, we have observed that decreased long chain CER levels following LSG is likely to contribute to the rapid decrease of insulin resistance observed in bariatric patients post-operatively. Referenced studies and the findings reported herein reinforce that circulating ceramide levels may be an important mediator of insulin sensitivity in peripheral tissues. The restoration in insulin sensitivity, probably secondary to reduced ceramide levels, emphasize one of the most important benefits of LSG in reducing comorbidities associated with obesity. It provides additional evidence to support the development and application of surgical techniques for the treatment of obesity.

## Abbreviations

ALP: Alkaline phosphatase
BMI: Mean body mass index
C1P: Ceramide-1-phosphate
CER: Ceramide
CerS: Ceramide synthase
CPK: Creatine phosphokinase
CVD: Cardiovascular disease
ELISA: Enzyme-linked immunosorbent assay
EWL: Excess weight loss
FFA: Free fatty acid
GLP-1: Glucagon-like peptide 1
Hb: Hemoglobin
HbA1c: Hemoglobin A1c
HDL-C: High-density lipoprotein cholesterol
HOMA IR: Homeostatic model assessment for insulin resistance
IL-1: Interleukin-1
LDL-C: Low-density lipoprotein cholesterol
LSG: Laparoscopic sleeve gastrectomy
LSG: Laparoscopic sleeve gastrectomy
MRM: Multiple reaction monitoring
MS/MS: Tandem mass spectrometry
NLRP3: Nucleotide-binding domain, leucine-rich-containing family, pyrin domain-containing 3
N-SMase: Neutral sphingomyelinase
*PKB*: *Protein kinase B*
PYY: Peptide YY
SM: Sphingomyelin
SPH: Sphingosine
SPT: Serine palmitoyltransferase
T2DM: Type 2 diabetes mellitus
TC: Total cholesterol
TG: Triacylglycerol
TNF-α: Tumor necrosis factor
TSH: Thyroid-stimulating hormone
UFLC: Ultra fast-liquid chromatography
VLDL-C: Very low-density lipoprotein cholesterol
